# NGR (Asn-Gly-Arg)-targeted delivery of coagulase to tumor vasculature arrests cancer cell growth

**DOI:** 10.1038/s41388-018-0213-4

**Published:** 2018-04-17

**Authors:** Khaled Seidi, Rana Jahanban-Esfahlan, Hassan Monhemi, Peyman Zare, Babak Minofar, Amir Daei Farshchi Adli, Davoud Farajzadeh, Ramezan Behzadi, Mehran Mesgari Abbasi, Heidi A. Neubauer, Richard Moriggl, Nosratollah Zarghami, Tahereh Javaheri

**Affiliations:** 1 Department of Medical Biotechnology, Faculty of Advanced Medical Sciences, Tabriz University of Medical Sciences, Tabriz, Iran; 2 Department of Chemistry, University of Neyshabour, Khorasan Razavi Province, Neyshabour, Iran; 3 Department of Pathobiology, Faculty of Veterinary Sciences, University of Tabriz, Tabriz, Iran; 4 Center for Nanobiology and Structural Biology, Institute of Microbiology, Academy of Sciences of the Czech Republic, Zámek 136, 373 33 Nové Hrady, Czech Republic; 5 Faculty of Science, University of South Bohemia, Branišovská 1760, 37005 České Budějovice, Czech Republic; 6 Department of Cellular and Molecular Biology, Faculty of Biological Science, Azarbaijan Shahid Madani University, Tabriz, Iran; 7 North Research Center, Pasture Institute of Iran, Amol, Iran; 8 Drug Applied Research Center, Tabriz University of Medical Sciences, Tabriz, Iran; 9 Ludwig Boltzmann Institute for Cancer Research, 1090 Vienna, Austria; 10 Institute of Animal Breeding and Genetics, University of Veterinary Medicine Vienna, 1210 Vienna, Austria; 11 Medical University of Vienna, 1090 Vienna, Austria; 12 Department of Clinical Biochemistry and Laboratory Medicine, Faculty of Medicine, Tabriz University of Medical Sciences, Tabriz, Iran; 13 Iranian National Science Foundation, Tehran, Iran

## Abstract

Induction of selective thrombosis and infarction in tumor-feeding vessels represents an attractive strategy to combat cancer. Here we took advantage of the unique coagulation properties of staphylocoagulase and genetically engineered it to generate a new fusion protein with novel anti-cancer properties. This novel bi-functional protein consists of truncated coagulase (tCoa) and an NGR (GNGRAHA) motif that recognizes CD13 and α_v_β_3_ integrin receptors, targeting it to tumor endothelial cells. Herein, we report that tCoa coupled by its C-terminus to an NGR sequence retained its normal binding activity with prothrombin and a_v_β_3_ integrins, as confirmed in silico and in vitro. Moreover, in vivo biodistribution studies demonstrated selective accumulation of FITC-labeled tCoa-NGR fusion proteins at the site of subcutaneously implanted PC3 tumor xenografts in nude mice. Notably, systemic administration of tCoa-NGR to mice bearing 4T1 mouse mammary xenografts or PC3 human prostate tumors resulted in a significant reduction in tumor growth. These anti-tumor effects were accompanied by massive thrombotic occlusion of small and large tumor vessels, tumor infarction and tumor cell death. From these findings, we propose tCoa-NGR mediated tumor infarction as a novel and promising anti-cancer strategy targeting both CD13 and integrin α_v_β_3_ positive tumor neovasculature.

## Introduction

Tumor infarction mediated by targeted delivery of coagulating proteins represents an appealing and cost effective strategy to combat a broad range of cancers [1]. Since 1997, studies have described the use of a truncated form of the human coagulation-inducing protein tissue factor (tTF) conjugated to diverse tumor endothelial moieties such as NGR (Asn-Gly-Arg) peptides. Notably, such fusion proteins have been utilized for specific induction of thrombosis in the neovasculature of tumors in various animal models [2–10]. So far, tTF-NGR remains the only drug of this nature to be tested in a phase I clinical trial [2]. Systemic injection of 1–4 mg/m^2^ doses of tTF-NGR in patients with advanced cancer was well tolerated and shown to selectively inhibit tumor perfusion as verified by measuring the vascular volume fraction (VVF) [2].

Sequences containing an NGR motif recognize and bind CD13 on tumor endothelial cells [11, 12]. Furthermore, NGR deamidation produces an additional recognition moiety called Iso-DGR, which targets α_v_β_3_ integrin on the tumor neovasculature [13]. We recently published a similar approach utilizing RGD peptides that recognize only α_v_β_3_ integrin, but tumor targeting required a higher dose escalation [14]. Here we have further developed this tumor-specific neovascular targeting approach by using bi-specific NGR peptides that recognize both CD13 and α_v_β_3_ integrin, in order to lower the dose escalation and achieve more physiologic targeting of tumor neovasculature to avoid potential negative side effects.

CD13 and α_v_β_3_ integrins are integral angiogenesis regulators, and they are overexpressed on the endothelium of solid tumors where they help promote tumor vascularization [15, 16]. Moreover, CD13 and α_v_β_3_ integrins play an essential role in metastasis; these receptors are only expressed in newly formed vessels, facilitating a developmental angiogenesis program not present in resting endothelial cells and most normal organ systems in adults [13, 17, 18]. These characteristics make them a useful cell-surface target for anti-angiogenic therapy. Therefore, owing to its low molecular weight, ease of synthesis, and selective affinity for tumor neovasculature cellular targets, NGR represents a favorable tumor-homing motif for targeted cancer therapy [11]. Although NGR-directed delivery of tTF results in the selective induction of tumor infarction, its clinical application is currently limited due to incomplete induction of thrombosis as well as side effects in treated animals [1, 19].

Members of the *Staphylococcus* family of pathogenic gram-positive bacteria produce a novel clotting enzyme called staphylocoagulase (herein referred to as coagulase), which stimulates human blood coagulation systems [20]. A unique mechanism of prothrombin activation by coagulase, termed “molecular sexuality”, was proposed in 2003 by Friedrich et al. [21]. According to this mechanism, coagulase activates prothrombin by introducing conformational changes, as opposed to enzymatic digestion, which is described for zymogen activation by other proteins. Furthermore, it was reported that truncated coagulase (~325 aa) is sufficient for coagulase activity, whereas the N-terminal hexapeptide (Ilu1-Tyr6) is required for zymogen activation [21]. Coagulases are bifunctional proteins, as they bind to and activate prothrombin via N-terminal domain interactions, in addition to binding fibrinogen via 5–8 tandem 27-amino acid C-terminal repeat sequences [22, 23]. Coagulase binds to thrombin and prothrombin, leading to proteolytically active complexes that generate fibrin by cleaving fibrinogen, without cleaving other physiological thrombin substrates [21].

**Fig. 1:**
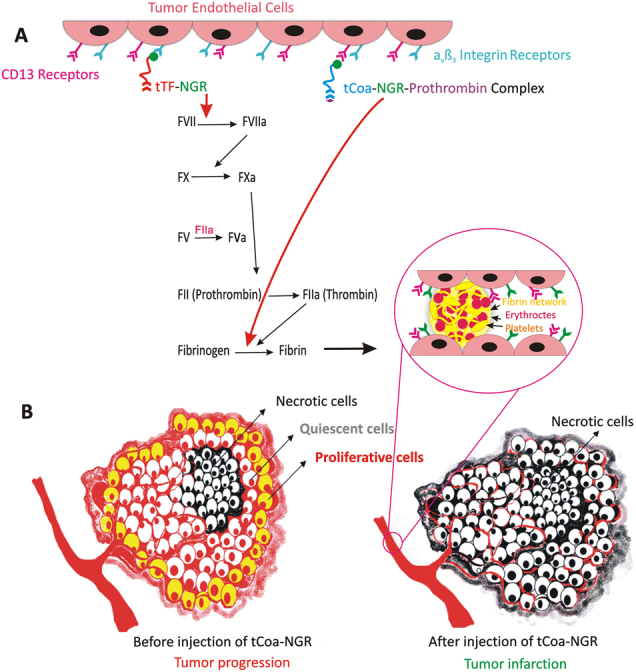
Differences between induction of thrombosis by NGR modified bacterial coagulase and human tissue factor. **a** Tissue factor (TF)-mediated thrombosis is systemic and involves stepwise activation of various clotting factors, while coagulase mediated coagulation is local and only affects fibrinogen levels. **b** Impact of tCoa-NGR on solid tumors. Before injection of the novel fusion protein, solid tumors are proliferative, harboring three different cell populations: highly proliferative cells in the tumor edge, necrotic cells in the core, and dormant cells in the middle layers. Treatment of solid tumors with tCoa-NGR results in the induction of thrombosis, occlusion of tumor-feeding blood vessels, tumor infarction, and oxygen, energy and nutrient depletion of the cancer cells undergoing anaerobic glycolysis, subsequently promoting necrosis of tumor cells. Part B of this figure is adopted from Jahanban-Esfahlan, R. et al. [1]

There are distinct differences in the mechanisms of coagulation induction between coagulase and tissue factor, which are schematically depicted in Fig. 1. Regular coagulation is accompanied by the formation of various coagulation factors, including tissue factor, FVII, FV, FX, platelets and thrombin, which can stimulate tumor growth and promote metastasis [24–26]. Notably, coagulase administered at only low doses acts upon fibrinogen, leaving all other clotting factors, including platelets, II, V, VIII, IX, X, XI and XII inactivated. Since coagulase bypasses the regular coagulation cascade and avoids formation of these additional clotting factors, its action is more locally restricted and does not induce an amplified systemic effect [27]. Furthermore, since coagulase binds to free thrombin, it acts as more of a scavenger of this protein [20]. Coagulase also has relatively poor antigenic properties, and at low doses, it does not produce any significant immunological response in mice [27]. Moreover, it has non-toxic enzyme activity that only elicits a coagulation response, without activating other pathways that promote angiogenesis [24]. Therefore, coagulase has various favorable thrombogenic properties and as such, it is of interest to explore the therapeutic potential of utilizing coagulase for cancer therapy.

Here we designed and generated a novel truncated form of coagulase bearing an NGR sequence on its C-terminus (tCoa-NGR). Accordingly, the 3D structure of the truncated enzyme was modeled and evaluated by homology modeling, molecular dynamics (MD) simulations, and docking studies. Functional studies demonstrated that tCoa-NGR could mediate coagulase activity, and the dual binding potential of His-tagged tCoa-NGR to CD13 and α_v_β_3_ integrin was confirmed by competition assays with NGR or RGD motifs. Furthermore, biodistribution studies using FITC-tagged tCoa-NGR verified tumor-specific accumulation of the fusion proteins in tumor bearing mice. Finally, systemic administration of tCoa-NGR in mice bearing 4T1 murine mammary or PC3 human prostate xenografts resulted in a striking reduction in tumor growth of both sex steroid-driven distinct tumor types. Notably, significant tumor cell growth arrest was accompanied by massive thrombotic occlusion of small and large tumor vessels, tumor infarction, followed by tumor necrosis. Overall, our data demonstrate the thrombogenic and therapeutic performance of tCoa-NGR in the induction of selective thrombosis and tumor infarction.

## Results

### In silico modeling, docking, and molecular dynamics simulation of tCoa-NGR and its interaction with prothrombin

**Fig. 2:**
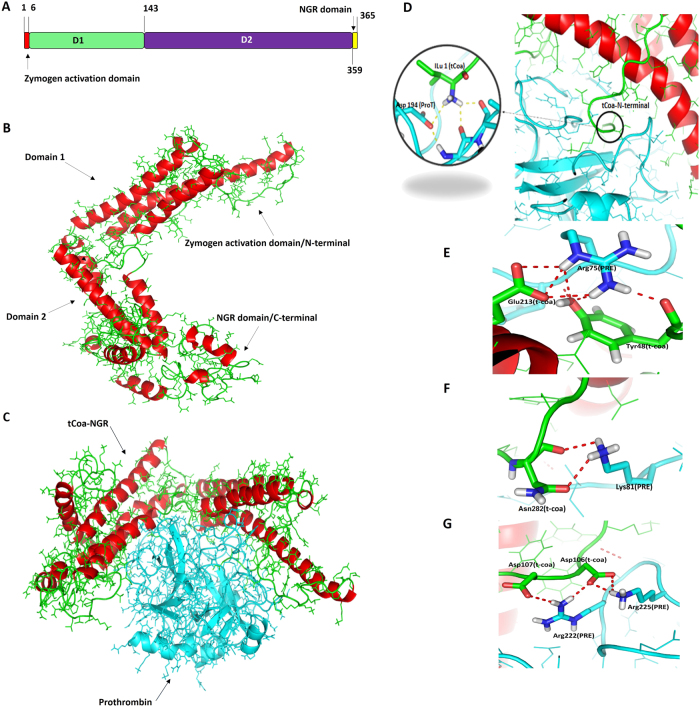
Molecular dynamic studies of tCoa-NGR proteins *i*n silico. **a** Protein structure of tCoa-NGR. **b** 3D structure of tCoa-NGR. Helical structure of tCoa-NGR within its two important domains. **c** Molecular dynamic simulation of tCoa-NGR-prothrombin complex. **d** Insertion of tCoa N-termius into prothrombin. **e** Interactions of Arg^75^ of prothrombin with Glu^213^ and Tyr^48^ of tCoa. **f** Interactions between Lys^81^ of prothrombin and Asn^282^ of tCoa-NGR. **g** Interactions between basic residues of prothrombin and acidic residues of tCoa-NGR

Prior to experimental studies, we assessed the predicted conformation of the novel tCoa-NGR fusion protein, and its interaction with prothrombin, through in silico molecular dynamics (MD) analyses. The structural domains of tCoa-NGR are graphically depicted in Fig. 2a, and the 3D structure of tCoa-NGR after homology modeling and MD simulation equilibration is illustrated in Fig. 2b. Furthermore, the 3D structure of tCoa-NGR in complex with prothrombin, after docking and MD simulation equilibration, is illustrated in Fig. 2c. Notably, the tCoa-NGR-prothrombin complex derived from our MD simulation closely resembles a previously reported complex of prothrombin and another staphylocoagulase variant [21], suggesting that our engineered fusion protein adopts a similar biologically-relevant conformation.

The activation of the N-terminal zymogen is a crucial characteristic of the tCoa-NGR–prothrombin complex. As illustrated in Fig. 2d, the N-terminus of tCoa-NGR inserts into the activation cavity of prothrombin through the first hexapeptide Ile^1^ to Tyr^6^. This result is in agreement with zymogen activation via the postulated ‘molecular sexuality’ mechanism [21–23]. Moreover, there are many important structural interactions that occur between the proteins that provide complex stability and function (Fig. 2e–g). Interactions between Arg^75^ of prothrombin, and Glu^213^ and Tyr^48^ of tCoa form strong interactions with multiple polar contacts (Fig. 2e). Similar residue interactions were reported between other coagulase variants in complex with prothrombin [21]. Additionally, interactions between Lys^81^ of prothrombin and Asn^282^ of tCoa-NGR (Fig. 2f), as well as the formation of multiple hydrogen bonds between basic residues of prothrombin and acidic residues of tCoa-NGR in the interaction interface of our model (Fig. 2g), predict a strong interaction between tCoa-NGR and prothrombin. Overall, these results indicate that our novel tCoa-NGR fusion protein is likely to form the expected targeted interaction with prothrombin.

### tCoa-NGR fusion proteins can activate prothrombin to induce coagulation and they specifically bind microendothelial cell receptors

**Fig. 3:**
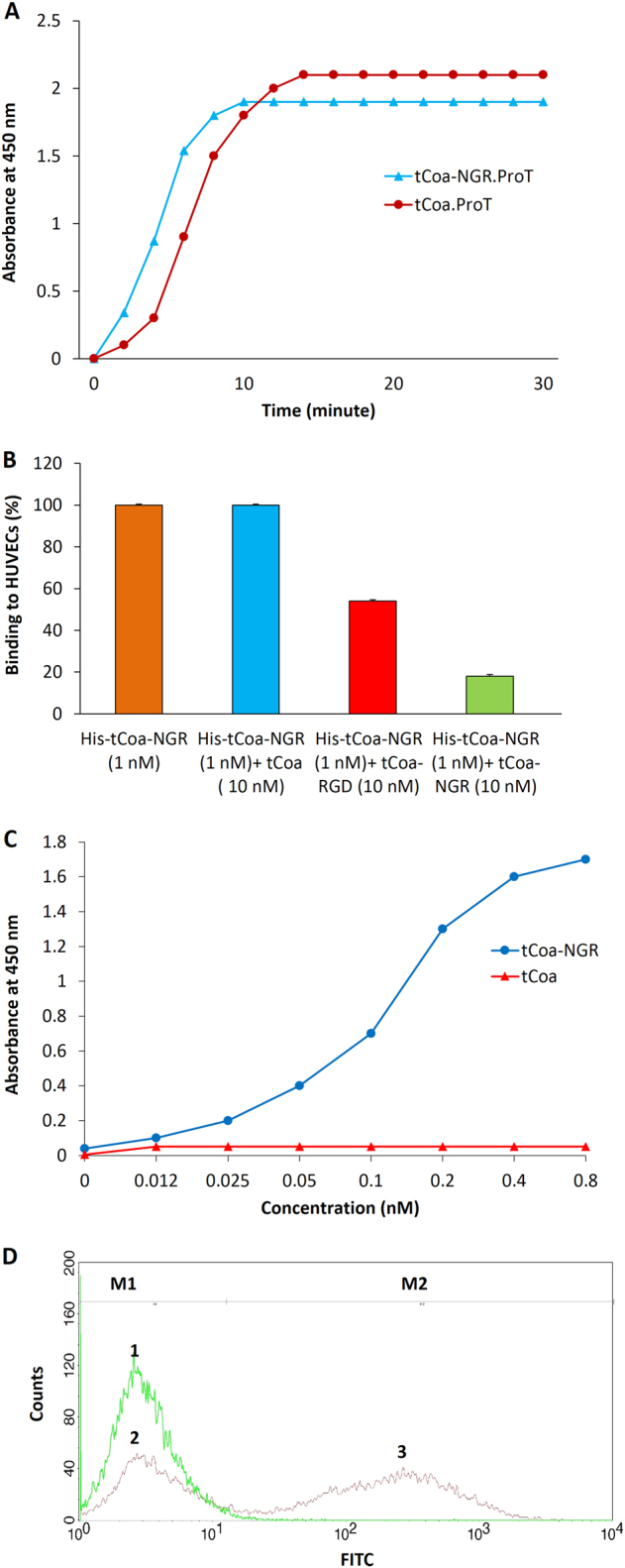
Functional studies to demonstrate coagulase activity and binding potential of tCoa-NGR fusion proteins in vitro. **a** Induction of thrombosis was measured to demonstrate tCoa clotting activity by tCoa-NGR fusion proteins. tCoa-NGR was capable of activating prothrombin at 1:1 nanomolar concentrations, comparable to that of prothrombin activation by tCoa at each time point. **b**–**d** Ligand-receptor binding studies to demonstrate binding of tCoa-NGR to CD13 and α_v_β_3_ integrin by ELISA and FACS. **b** Addition of non His-tagged tCoa-RGD (α_v_β_3_ integrin receptor inhibitor) or tCoa-NGR (α_v_β_3_ and CD13 receptor inhibitor) as competitive ligands of NGR motif, resulted in 46% and 82% binding inhibition of His-tagged tCoa-NGR to its endothelial receptors, respectively. **c** tCoa-NGR but not tCoa specifically binds to immobilized endothelial cells in a dose-dependent fashion, with the highest binding at 0.8 nM concentration. **d** Differential binding of tCoa (peak 1) and tCoa-NGR (peak 2, 3) to endothelial cells in suspension was assessed by FACS. HUVECs were incubated with either tCoa or tCoa-NGR, and proteins that bound to the cells were FITC-labeled and detected by FACS. In this histogram, the M1 marker identifies cells that were not bound by proteins, while the M2 marker comprises the cells positive for bound proteins

Following the generation, purification and validation of tCoa and tCoa-NGR (Supplementary Fig. S1), functional studies were performed to assess their coagulase activity, through the ability to activate prothrombin and induce blood coagulation. Indeed, upon mixing the proteins with prothrombin at a 1:1 ratio to generate protein complexes, both tCoa-NGR and tCoa were able to activate prothrombin in a time-dependent manner, as assessed by the conversion of fibrinogen to fibrin (Fig. 3a). Furthermore, after incubation of sodium citrate-treated mouse blood with tCoa-NGR, tCoa or PBS, it was found that the addition of either tCoa-NGR fusion protein or tCoa effectively enhanced blood coagulation within 30 min (28 min for tCoa-NGR and 25 min for tCoa; data not shown). Importantly, incubation with PBS alone did not result in blood coagulation (data not shown). Therefore, these data indicate that the addition of the NGR sequence to the C-terminus of tCoa does not hinder its coagulation activity.

Next, the specific binding of tCoa-NGR with endothelial cell markers was assessed, to validate the use of the NGR peptide as an endothelium-homing motif. Selective binding of His-tagged tCoa-NGR to CD13 and α_v_β_3_ integrins expressed on HUVECs was determined by ELISA assays. The specific interaction between the NGR motif and both CD13 and integrin α_v_β_3_ was confirmed by competitive binding assays using non His-tagged tCoa-NGR or tCoa-RGD, a control fusion protein that acts as a competitive ligand to integrin α_v_β_3_ but not CD13. Our results demonstrated that 10-fold molar excess of tCoa-NGR resulted in >80% binding inhibition of His-tCoa-NGR to HUVECs (Fig. 3b). In contrast, 10-fold molar excess of tCoa as a negative control showed no inhibitory effect on His-tCoa-NGR binding, indicating the high specificity of NGR-modified coagulase to the endothelial cells. Furthermore, the specific interaction of tCoa-NGR with CD13 was assessed in the presence of 10-fold molar excess tCoa-RGD. Correspondingly, we observed only a ~46% decrease in binding of His-tagged tCoa-NGR in the presence of the RGD motif, which was considerably lower than that induced by NGR competitive binding (Fig. 3b), which is consistent with RGD competing for binding to integrin α_v_β_3_ but not CD13.

Further, we validated that tCoa-NGR was able to interact with endothelial cell receptors in a dose-dependent manner at low concentrations, with the strongest binding activity occurring at 0.8 nM. In contrast, no binding was detected between HUVECs and tCoa, lacking the NGR motif at these concentrations (Fig. 3c). Moreover, binding of tCoa-NGR to endothelial cell receptors was also assessed by FACS. As presented in Fig. 3d, FITC-labeled tCoa-NGR showed a ~60% binding efficiency to endothelial cells in suspension, whereas there was no detectable signal for cells incubated with FITC-labeled tCoa. Together, these data validate the importance of the NGR motif for endothelial cell-target binding and highlight the dual endothelial receptor-targeting ability of the NGR moiety of our fusion protein.

### tCoa-NGR fusion proteins accumulate at the tumor site in xenografted mice

**Fig. 4:**
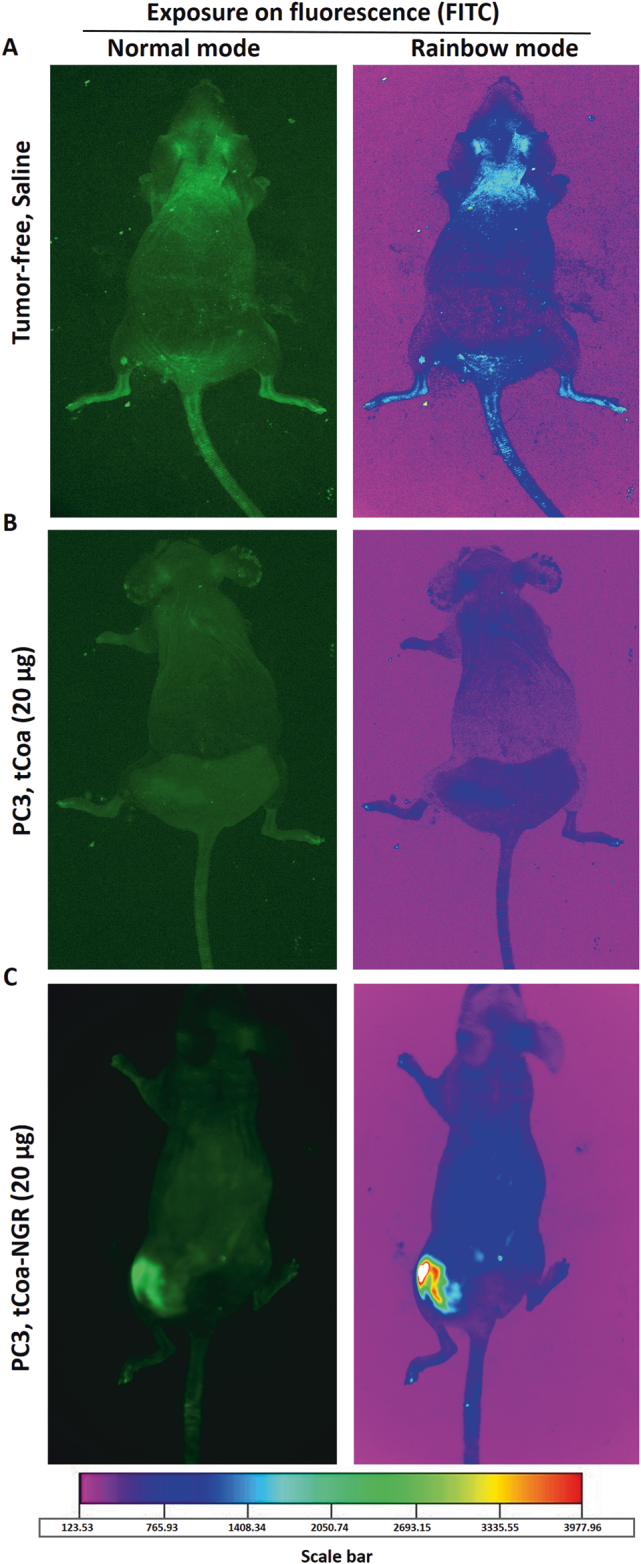
Tracing fluorescently labeled tCoa-NGR proteins in vivo. **a** Tumor-free mice injected with saline as control. Mice bearing PC3 prostate cancer xenografts were injected with either (**b**) tCoa labeled with FITC or (**c**) tCoa-NGR labeled with FITC (*n* = 6). Selective accumulation of targeted fusion protein to tumor neovasculature was assessed by an in vivo imaging system

PC3 tumor xenografts express high levels of CD13 and α_v_β_3_ integrin receptors, which are absent from the vasculature of normal tissues. Therefore, to assess the biodistribution and tumor-homing properties of tCoa-NGR in vivo, FITC-labeled proteins were injected in C57BL/6 nude mice bearing PC3 xenografts, and were monitored by live imaging. As shown in Fig. 4a, tumor-free mice injected with saline produced no considerable background fluorescence or specific accumulation in any organs of these healthy control mice. Likewise, no observable fluorescence enhancement was detected at the site of subcutaneously transplanted PC3 xenografts in mice that were injected with FITC-labeled tCoa (Fig. 4b). Notably, FITC-labeled tCoa-NGR was strongly and selectively accumulated at the site of the PC3 xenografts, but not within any other tissues of the mice (Fig. 4c). In line with our in vitro binding studies, these results demonstrate selective and specific NGR-directed delivery of the fusion protein to the tumor neovasculature.

### In vivo administration of tCoa-NGR fusion proteins reduces tumor growth by inducing thrombosis

**Fig. 5:**
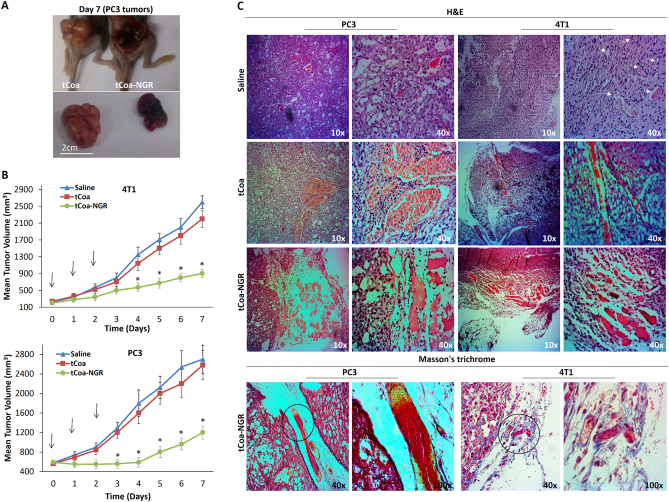
Therapeutic potential of tCoa-NGR proteins in vivo. **a** ​Illustrative photos of mice bearing prostate cancer xenografts (PC3) at the end of treatment (day 7) treated with tCoa-NGR (right) or tCoa (left). **b** 4T1 and PC3 tumor-bearing mice were injected intravenously with saline, 10 µg tCoa or 10 µg tCoa-NGR (*n* = 6). **c** Histological analysis of 4T1 mouse mammary xenografts in BALB/c mice and PC3 human prostate carcinoma xenografts in C57Bl/6 nude mice treated with either saline, tCoa or tCoa-NGR fusion proteins. Arrows represent intact vessels throughout tumor tissue sections in mice treated with saline. Conversely, tCoa-NGR tumor tissues presented occluded blood vessels with packed erythrocytes and fibrin clots, indicating an induction of thrombosis in the vasculature of 4T1 and PC3 tumors. Several thrombosed blood vessels are selected, and the same location is shown with two different magnifications for clarity. Moreover, induction of complete thrombosis in the neovasculature of mice bearing 4T1 or PC3 tumors was explored through distinct staining of fibrin (red) and red blood cells (yellow) by Masson’s trichrome staining

In order to assess the reproducibility of obtained results, the therapeutic performance of tCoa-NGR fusion protein was evaluated in two animal models: immune-competent BALB/c mice bearing murine 4T1 mammary carcinoma allografts, and human PC3 prostate cancer xenografts in immune-compromised C57BL/6 nude mice. Mice bearing either 4T1 or PC3 tumors were each administered with either 10 µg tCoa-NGR fusion protein, or tCoa control, daily for three consecutive days, and tumor progression was measured up to seven days. Our results indicated macroscopic evidence of thrombosis and hemorrhaging at the site of the subcutaneously implanted PC3 tumor xenografts treated with tCoa-NGR fusion protein, while no thrombotic changes were detected in the tumor xenografts of mice that were treated with tCoa (Fig. 5a), suggesting that tCoa-NGR effectively mediated selective blood coagulation in the tumor vasculature. Also, administration of tCoa-NGR resulted in significantly reduced tumor growth compared with administration of tCoa or saline, in both 4T1 and PC3 tumor models (Fig. 5b).

In addition to these macroscopic observations, the significantly smaller tumor volume in the tCoa-NGR treated group was accompanied by microscopic markers of thrombosis and necrosis within the tumors, as confirmed by histo-pathology analysis. Specifically, H&E staining showed viable tumor cells and healthy and intact blood vessels in the group injected with either saline or tCoa (Fig. 5c). In the group treated with tCoa-NGR, however, early signs of thrombosis with aggregated erythrocytes and mesh networks of fibrin were detected in almost all tumor blood vessels following 24 h after the injection. Furthermore, induction of complete thrombosis in the neovasculature of 4T1 and PC3 tumors treated with tCoa-NGR was confirmed by Masson’s trichrome staining (Fig. 5c). Interestingly, both small and large blood vessels of 4T1 and PC3 solid tumors displayed efficient and complete thrombosis and collapse of tumor neovasculature, indicating that the efficient induction of thrombosis by tCoa-NGR fusion proteins can be a successful anti-cancer strategy.

**Fig. 6:**
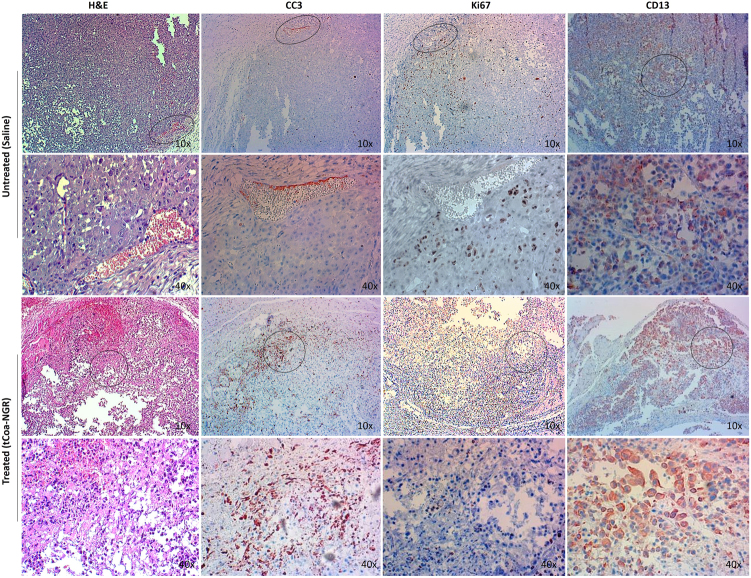
H&E and immunohistochemistry analysis of PC3 tumor sections stained with CD13, Ki67 and CC3 in saline controls and groups treated with tCoa-NGR fusion proteins

Additionally, to explore the molecular mechanisms behind the potent anti-tumor activity of tCoa-NGR proteins, histological sections of the PC3 tumors were stained to examine levels of cleaved caspase 3 (CC3), Ki67 and CD13 expression. As shown in Fig. 6, tCoa-NGR-treated animals had significantly increased levels of CC3 within the tumors as compared to control saline treated animals, indicating an induction of tumor cell apoptosis. Furthermore, tumors from control mice were positive for the proliferation marker Ki67, whereas Ki67 staining was considerably decreased in tumors from the tCoa-NGR treated group (Fig. 6), which is in line with the significant reduction in tumor growth observed in these mice (Fig. 5a, b). Moreover, CD13 staining was positive in the PC3 tumor sections from both tCoa-NGR treated and control mice (Fig. 6). This was expected as the PC3 prostate cancer line has high expression of CD13, making it an ideal therapeutic target for NGR-directed delivery of truncated coagulase to efficiently induce thrombosis, reduce tumor cell proliferation and increase cell death in these highly aggressive and widely used human tumor cell line models.

### tCoa-NGR fusion proteins are well tolerated at higher doses in vivo

**Fig. 7:**
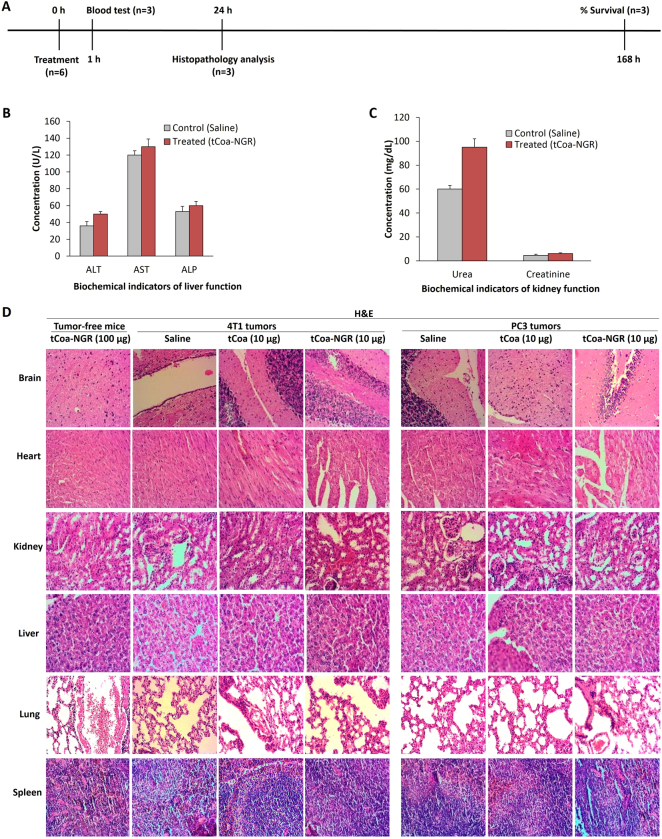
Toxicological/drug tolerability studies in mice. **a** Timing schedule of toxicological experiments in healthy mice injected with a high dose (100 μg) of tCoa-NGR or saline. **b**, **c** Serological and biochemical analysis of healthy mice injected with a high dose of tCoa-NGR or saline. **d** H&E staining of organs (brain, heart, kidney, liver, lung and spleen) from the cancerous mice treated with saline, tCoa (10 µg), and tCoa-NGR (10 µg) to demonstrate toxicity profiles of the fusion proteins compared to the control (saline). Histological sections of normal organs from healthy mice injected with a high dose of tCoa-NGR fusion proteins (100 µg) is also presented to demonstrate drug tolerability. Magnification (×40). AST aspartate transaminase, ALP alkaline phosphatase, ALT alanine aminotransferase

The drug toxicity/tolerability of tCoa-NGR fusion proteins in vivo was further examined by injection of a single higher dose of tCoa-NGR (100 µg), to rule out potential toxic side effects. We monitored injected animals by macroscopic examination (such as atypical bleeding, tail necrosis, unexpected death and weight loss), serological testing of blood biochemical indicators, as well as microscopic analysis (H&E staining) (Fig. 7a). All mice injected with a single high-dose of tCoa-NGR, or saline, were alive after 1 week post administration and displayed no obvious adverse side-effects (data not shown). Moreover, biochemical indicators of liver enzymes, including aminotransferase levels and liver damage parameters such as AST, ALT, ALP were at normal levels and comparable to control mice (Fig. 7b). Likewise, biochemical indicators of kidney filtering function, including urea and creatinine levels were measured. In line with normal liver function, kidney function as indicated by creatinine levels was unchanged, whereas urea concentrations were moderately increased (~1.5 fold) in tCoa-NGR-treated mice compared with saline treated mice (Fig. 7c), indicating that a high dose of tCoa-NGR at 100 µg might start to impair normal kidney function.

Furthermore, histological analysis of organs from the two established mouse tumor models receiving multiple lower doses (10 µg) of saline, tCoa or tCoa-NGR showed no signs of thrombosis or necrosis in normal tissues, including spleen, brain, lung, kidney, heart, and liver (Fig. 7d). Likewise, histological examination of organs from mice injected with a single high dose of tCoa-NGR revealed no signs of thrombosis or obvious organ damage after 24 h (Fig. 7d). Overall, our study demonstrates high in vivo safety and target-selectivity of the tCoa-NGR fusion proteins.

## Discussion

To specifically target the tumor neovasculature in conjunction with the tumor cells themselves is an appealing strategy to combat cancer cell growth, particularly as demonstrated here by targeting more than one single angiogenesis marker [28]. As such, we took advantage of the dual targeting potential of the NGR moiety (GNGRAHA), as a selective tumor endothelial homing tag, to target coagulase to CD13 and α_v_β_s_ integrin, which are abundantly and predominantly expressed on the angiogenic endothelium of solid tumors [13]. The dual targeting potential of the NGR motif permitted efficient induction of thrombosis in the neovasculature of 4T1 and PC3 tumors in mice with very low concentrations of NGR-coagulase fusion proteins. This novel treatment resulted in subsequent tumor infarction, and a significant reduction in the growth of mammary and prostate solid tumors in vivo.

Since both CD13 and α_v_β_s_ integrin signaling and associated tumor neovasculature formation are important for metastatic dissemination, one could also postulate a beneficial therapeutic effect of this therapy in blocking cancer cell tissue invasion [12]. Angiogenesis and the formation of new blood vessels is a prerequisite for the survival, growth and also metastasis of tumor cells, and it is also linked to cancer metabolism [29]. This is particularly the case in association with the Warburg effect and anaerobic glycolysis through lactate production, triggering HIF1 alpha activation. Tumor cells are often embedded deeply within the tumor stroma and can therefore evade chemotherapeutic agents and even immune cells [30]. Compromising the tumor vasculature could also lead to better penetration of immune cells or drug uptake into tumor cells [31]. Vascular targeting agents are applicable for the treatment of almost all types of tumors, especially those presenting a highly proliferative and invasive phenotype associated with dense vessel formation [19]. These agents fall into the following three main groups: (1) those that inhibit angiogenesis, (2) those that disrupt tumor vasculature, and (3) those that induce tumor vascular infarction [32]. Although many angiogenesis inhibitors are FDA approved, unfortunately their application in the clinic has demonstrated only moderate benefits in increasing the standard of care and lifespan of patients, generally due to the development of drug resistance [33]. Furthermore, costly targeted cancer therapeutics have limits in less financed health care systems, whereas our approach could have a significant advantage here since it is cost effective to produce and monitor the clinical action of NGR-coagulase fusion proteins.

While angiogenesis inhibitors aim to halt new blood vessel growth, tumor vascular disruption and tumor vascular infarction work through the disruption and occlusion of already formed blood vessels. This causes a hypoxic environment, and rapid ischemia, necrosis, and death of the tumor bulk can be achieved [34]. Vascular targeting agents such as DMXAA and Combretastatin A4 phosphate, and their derivatives, have been extensively evaluated in preclinical and clinical settings [31]. Nonetheless, TF-NGR remains the only drug with the capacity to induce tumor vascular collapse associated with a marked reduction in tumor growth, and it is currently being tested in a phase I clinical trial (NCT02902237) [2]. It is noteworthy that, to date, neither angiogenesis inhibitors nor agents that target blood vessels provide a curative potential unless combined with other modalities, including chemo-, radio-, radioimmuno-, photodynamic-, or even infectious disease therapy [19].

The fact that anti-angiogenesis and tumor vascular disruption monotherapies show no curative potential underpins the clinical significance of tumor vascular infarction as a single therapy capable of ablating large tumors in mice, as reported by several groups [2, 5, 6, 9, 35]. The first coaguligand (clotting factor plus an endothelial targeting tag) displayed efficacy in inducing selective tumor vascular infarction in neuroblastoma xenografts [6]. In this study, a bispecific antibody composed of tTF and an antibody against MHC-II as an artificial marker of angiogenesis was injected into mice bearing neuroblastomas. Upon a single injection, massive thrombosis occurred throughout the tumor within 30 min, and extensive intravascular thrombosis of the tumor lasted for more than 3 days, resulting in complete regressions (CRs) in 38% of the treated mice, which persisted for up to 4 months and longer [6]. Furthermore, targeted tTF to the EDB domain of fibronectin on the vasculature of three different types of solid tumors was efficacious [9]. The anti-tumor effect of a single injection (20 µg) of scFv(L19)-tTF was more dramatic in mice bearing larger tumors [9]. Higher doses of the fusion protein (35 µg) facilitated removal of the residual tumor mass and led to CR in ~30% of treated mice [9].

Subsequently, a coaguligand containing vascular endothelial growth factor (VEGF) and tTF was engineered (rVEGF-TF) [5]. Injection of two doses of 20 µg rVEGF-TF in mice bearing CT26 colon carcinoma xenografts resulted in a significant decrease in tumor growth and complete regression of tumor mass in 20% of mice by 2 weeks after the first treatment [5]. Furthermore, it was reported that the fusion of a short NGR motif to the C-terminus of tTF displayed anti-tumor-targeting capacity [2]. Specifically, multiple injections of 30 µg doses of tTF-NGR (3–6 times, i.v) in mice xenografted with established human adenocarcinoma (A549), melanoma (M21), or fibrosarcoma (HT1080) resulted in a significant growth inhibition in all three solid tumor models. Furthermore, when using higher, more toxic doses (3–5 mg/kg, s.c), complete remissions were accomplished in a small number of animals with no relapse of tumors even after prolonged observation [2]. Importantly, we did not see side effects in the present study, but systemic toxicity with tTF-NGR, including necrosis of mouse tail tip, DIC, pulmonary embolism, and death was reported with both subcutaneous and intravenous administration with LD10 (lethal dose for 10% of the animals) ≥5 mg/kg, i.v [2, 36].

The results of our recent study employing a truncated form of coagulase bearing an RGD (GRGDSP) motif on its C-terminus showed that tCoa-RGD therapy resulted in augmented thrombosis and a significant growth regression of three established solid tumor models in mice, with 4T1 mouse mammary tumors as a high growth rate model and CT26 mouse colon and SKOV3 human ovarian carcinoma cells resulting in medium growth rate tumors [14]. Administration of tCoa-RGD fusion proteins (15 µg × 3q 24 hours) caused sustained induction of thrombosis and almost completely inhibited the growth of rapidly-growing 4T1 tumors by day 5 after the first treatment. Compared to human tissue factor, the mechanism of coagulase from *Staphylococcus aureus* is quite unique, as it initiates blood coagulation via conformational activation of prothrombin. This non-enzymatic process leaves all clotting factors intact, with the exception of fibrinogen. Thus, coagulase bypasses the regular coagulation cascade and elicits local effects. Indeed, we observed a low effective-dose as well as a wide therapeutic window of coagulase fusion proteins, including tCoa-RGD [14] and tCoa-NGR (this study), in various xenograft tumor models, where large tumors with neovasculature were established.

It is reported that coagulase at concentrations as low as 1 × 10^−16^ M is sufficient for thrombin activation in vitro [37]. Compared to coagulase, tissue factor fusion proteins demand higher concentrations, equal to or more than 1 µmol/L [38]. In our study, nanomolar concentrations sufficed to initiate coagulation of blood. Moreover, compared to our experiments with tCoa-RGD [14], in this study, lower concentrations of tCoa-NGR (10 µg vs. 15 µg) were found to be optimal for induction of complete thrombosis in the neovasculature of two highly proliferative 4T1 and PC3 tumor models. This finding could be explained by the ability of the NGR motif to target multiple endothelial receptors such as CD13 and integrin α_v_β_3_. Simultaneous targeting of coagulase to two prominent angiogenesis makers via the NGR moiety resulted in efficient thrombotic activity of tCoa-NGR in the established tumors. Vascular thrombosis and tumor infarction were characterized by the formation of mesh networks of fibrin clots and reduced proliferation and survival of tumor cells, as validated by increased levels of CC3 and diminished Ki67 staining in histological tumor section of mice that were treated with tCoa-NGR. Considering the fact that rabbit and human blood are more sensitive to coagulase than mouse blood [27], it is predicted that targeted coagulase therapy in human applications would require even lower doses than utilized here in mice. These findings could be of clinical significance to reinforce safety profiles for coagulase treatment in cancer patients.

Given that coagulase is a non-human derived protein, there are some concerns regarding its neoantigen/immunogenicity and whether it provokes allergic sensitization in humans. However, the low concentration and limited duration of treatment required may limit any potential immunogenic action. Coagulase is a weak antigen with respect to eliciting systemic effects, as it was demonstrated that doses of 3.9 mg per patient of free coagulase given repeatedly to humans by various routes of administration (intravenous, intramuscular, and subcutaneous) were very well tolerated [27]. Defibrination and thrombosis could be considered as the main concerns surrounding the safety of coagulase, as intravenous injection of very large doses of coagulase (10 mg) in rabbits cause a marked and sudden drop in fibrinogen with widespread intravascular clotting, affecting mainly the lungs, followed by rapid death within 30 min [39]. Defibrination is the only complication that is reported with high doses of coagulase in rabbits [27], but this was not reported in mice (2 mg) or in humans (3.9 mg). Accordingly, in our study, we considered the most critical time for drug toxicity/tolerability to be within 24 h after injection of coagulase in mice. Thus, we assessed the mice for any side effects of coagulase injection at 1 h after the injection (serological analysis of murine blood) as well as histo-pathological analysis of vital organs at 24 h after treatment. Nonetheless, administration of higher doses of targeted coagulase (100 µg) resulted in no detectable toxicity or negative side effects. We did not observe any excessive weight loss, abnormal bleeding, diarrhea, liver or kidney malfunction, or any unexpected death in the animals at this dose.

An additional potential complication surrounding the application of clotting factors for cancer therapy is the risk of early and off-target induction of coagulation in the bloodstream or in other organs such as the lung [1]. Given the unique mechanism and local effects of coagulase described earlier [21, 27], as well as the ‘quorum acting’ mechanism of bacteria [40], it appears that a certain amount of coagulase molecules are required to cluster together to reach a ‘local optimum threshold concentration’ to initiate blood coagulation (Supplementary Fig. S2.A). In support of this, coagulase failed to stop bleeding caused by a deficiency in a certain clotting factor, despite the fact that prothrombin was present and the conversion of fibrinogen to fibrin could be initiated [27]. Indeed, directing coagulase to tumor endothelial cells via tumor-targeting moieties would provide proper localization of coagulase proteins in sufficient concentrations to efficiently and locally initiate thrombosis of tumor blood vessels. Though quorum acting is not yet validated for *Staphylococcus aureus*, we postulate that the observation of incomplete thrombosis in some histological tumor sections (Supplementary Fig. S2.B) is likely due to insufficient local optimum threshold concentrations at these regions. Accordingly, tumor target optimization (amount and distribution of receptors) appears to be the key to achieving optimum threshold concentrations of targeted coagulase, and will therefore be an important factor in the development of more robust anti-cancer drug strategies in the future.

Overall, we propose tCoa-NGR mediated tumor infarction as a novel and promising anti-cancer strategy to specifically target tumor neovasculature.

## Materials and methods

### Cell lines, bacteria strain, mice and reagents

The Pasteur Institute of Iran (Tehran and Amol) provided *Staphylococcus aureus* (ATCC 29213), mouse mammary carcinoma (4T1), human prostate adenocarcinoma (PC3), and human umbilical vein endothelial cells (HUVEC), as well as C57BL/6 nude mice. Mycoplasma testing of all cells was regularly performed. The *pet 28-a* expression vector was obtained from the Iranian Biological Resource Center (IBRC), Tehran. The following antibodies were used in this study: Anti poly Histidine-HRP (Sigma, #A7058), FITC-conjugated anti-6 × His-tag antibody (Abcam, #ab1206), Ki67 (Novocastra, NCL-KI-67-P), cleaved caspase 3 (Cell Signaling, #9661), and CD13 (Santa Cruz, #sc-136484).

### Molecular modeling, docking, and MD simulation

All bioinformatics studies were performed as previously described [14]. Briefly, the modeling of tCoa-NGR was performed by I-TASSER [41]. The interaction of tCoa-NGR with prothrombin (PDB: 1nu9 [21]) was studied by HADDOCK 2.2 [42]. All molecular schematics were constructed using PyMOL [43].

### Construction, cloning, expression, and purification of tCoa and tCoa-NGR fusion proteins

All procedures for manufacturing of recombinant proteins were performed as described in our previous work with tCoa-RGD [14], except for the following modifications. Regarding construct design, the previously described 18 bp sequence of RGD (GRGDSP) in the reverse primer was replaced with a 21 bp NGR coding sequence (GNGRAHA), generating the tCoa-NGR reverse primer: 5′-**GTACGCTCGAGTTA**TGCATGTGCTCTTCCGTTACC*TTGTAACGTTTTATTTTC*-3′. The sequence for NGR is underlined; the XhoI restriction site (CTCGAG) and a stop codon (TTA) was placed in the reverse sequence (bold); the italic area is the site for binding to the tCoa gene product.

### Blood clotting test

Blood coagulation tests were performed as previously described [14, 44]. Briefly, 1% sodium citrate was used to prepare citrated fresh mouse blood. Afterward, 5 µg purified tCoa-NGR or tCoa, or PBS alone as a negative control, was added to the tubes and incubated at room temperature. The presence of blood coagulation was documented at various time points by leaning the tubes at 45° angles.

### Coagulase activity assays

Coagulase activity was assessed as previously described [14, 44]. Briefly, human prothrombin (1% sodium citrate-PBS) was incubated with purified tCoa or tCoa-NGR (100 nM) at room temperature for 20 minutes. Next, the reaction was supplemented with 3 µM fibrinogen (Sigma), and the conversion of fibrinogen to fibrin was recorded every 2 min by measuring absorbance at 450 nm with a microplate reader (Biotek).

### Enzyme-linked immunosorbent assay (ELISA)

HUVECs expressing CD13 and α_v_β_3_ integrin receptors [2, 8] were cultured overnight in 96-well microplates in RPMI 1640 supplemented with 10% FBS at 37 °C, 5% CO_2_. After washing the wells with PBS, tCoa-NGR or tCoa was added to the wells in sequential nanomolar dilutions of 0, 0.25, 0.05, 0.1, 0.2, 0.4, and 0.8. In reference to our previous work [14], the absorbance for reaction was developed using an HRP-labeled anti-His tag antibody (Sigma).

For competitive binding, ELISA was conducted by addition of either non His-tagged tCoa-NGR fusion protein, as integrin α_v_β_3_ and CD13 antagonist, and non His-tagged tCoa-RGD fusion protein [14], as α_v_β_3_ integrin receptor antagonist only.

### Fluorescence-activated cell sorting (FACS)

FACS analysis was employed to verify the binding of tCoa-NGR to CD13/α_v_β_3_ integrin receptors, as previously described [14, 45, 46]. Briefly, human endothelial cells were mixed with either tCoa-NGR fusion proteins or tCoa (0.1 ng/10^6^ cells in ice-cold FACS buffer) for 60 minutes at room temperature under light-protected condition. Finally, after addition of FITC-conjugated anti-His tag antibody (Abcam) signals for FITC was captured using BD FACS Calibur.

### Mouse tumor models

All animal procedures were approved and conducted according to regulations of Laboratory Animal Ethics Committee of Tabriz University of Medical Sciences (license NO: 92/4-5/3) and in reference to our previous works [14, 45–49]. 4T1 (mouse mammary carcinoma) and PC3 (human prostate adenocarcinoma) tumor cell lines were grown to 70–80% confluence. Then, 5 × 10^5^ mammary or 5 × 10^6^ prostate cancer cells were injected subcutaneously into 3- to 5-week-old female BALB/c mice or C57BL/6 nude mice, respectively. The following formula was used to measure the tumor growth rate: volume (mm^3^) = length × width^2^ × 0.5.

### In vivo biodistribution of tCoa-NGR fusion proteins

Referring to our previous work [14], to assess the selective localization and binding of our fusion proteins in vivo, C57BL/6 nude mice bearing PC3 tumor xenografts were randomly divided into two groups of three mice each and injected with either 20 µg FITC-labeled tCoa-NGR or tCoa, through the tail vein. As a control, healthy mice without tumors were also injected with 200 µl saline through the tail vein. One hour later, accumulation of fluorescently conjugated fusion proteins was probed in vivo by KODAK imaging system (2000MM).

### Assessment of anti-tumor efficacy of tCoa-NGR in vivo

4T1 and PC3 xenografts were established as described above. When tumors with a volume of ~200–600 mm^3^ were established, mice were randomly distributed into three groups of six mice each. In one group, animals were intravenously administered a 10 µg dose of purified tCoa-NGR in 200 µl of PBS on the 1st, 2nd and 3rd day of the experiment. Employing the same schedule and methods, mice in another group were treated with 10 µg of purified tCoa, and mice in the last group were treated with 200 µl of PBS alone. 7 days after the first treatment, mice were killed.

### Histology and immunohistochemistry

To evaluate the selective induction of thrombosis 24 h after tCoa-NGR fusion protein (or tCoa control) treatment, normal organs and tumor tissues were collected for H&E and Masson’s trichrome staining [14, 47]. The induction of complete or incomplete thrombosis was evaluated according to the formation of fibrin mesh networks, observation of a blurred vessel outline, the presence of aggregated platelets, deposition of fibrin, and the extent of congested red blood cells [6].

Immunohistochemistry was performed as previously described [45–49] to assess CD13 localization and expression level, induction of apoptosis by CC3 staining, and the rate of proliferation by Ki67 staining. Analysis was performed on ‘day 7’ PC3 tumor sections from mice treated with tCoa-NGR or saline.

### Toxicity studies

For toxicity studies, organs of carcinoma bearing mice injected with saline, tCoa or tCoa-NGR were analyzed by H&E staining to detect signs of necrosis, thrombosis or hemorrhage. To test drug tolerability, tumor-free animals (healthy mice) were injected with a higher dose of tCoa-NGR (100 µg) or saline (*n* = 6). After 1 h, blood from three mice per group was collected to test serum levels of blood chemical markers for organ function. After 24 h, three animals per group were randomly selected and killed for histological analysis of normal organs, including brain, heart, lung, liver, spleen, and kidney. The survival time of the three remaining animals per group were documented for an additional 6 days.

### Statistical analysis and experimental validation

The data in our study are reported as mean ± SEs of usually three independent experiments, and statistical analyses were performed using SPSS version 16. To assess the statistical significance between independent groups, rank-sum test was employed and *P* < 0.05 was considered significant. For biological reproducibility and independent experimental validation, all experiments for histo-pathology analysis and in vivo studies were carried out at least twice.

## Electronic supplementary material

Supplementary figure legend

Figs. S1–S4
