# Supplementary material for: NGR (Asn-Gly-Arg)-targeted delivery of coagulase to tumor vasculature arrests cancer cell growth
Source: Oncogene. 2018 Apr 17;37(29):3967–80. doi: 10.1038/s41388-018-0213-4 (PMC6053358; doi:10.1038/s41388-018-0213-4)
Supplement: Supplementary file 1 — Supplementary figure legend [file 41388_2018_213_MOESM1_ESM.docx]

**Supportive data legend**

**Supportive Fig. 1.** Cloning, expression, and purification of tCoa-NGR fusion proteins. (A) Isolation of genomic DNA from *S. aureus.* (B) PCR amplification of full-length coagulase. (C) Double digestion of *pet* 28-a and PCR products. (D) Ligation of tCoa-NGR into the *pet* 28-a expressing vector. (E) Transformation of *pet* 28-a containing tCoa-NGR gene constructs into the *E. coli* *BL21* *(DE3)* host cells. (F) Colony PCR for detection of tCoa-NGR positive colonies using T7 universal primers. (G) Expression and purification of tCoa-NGR fusion proteins by NiNTA affinity chromatography and FPLC. Abbreviations: h (hour), M (molecular weight marker), E (elute), and W (wash).

**Supportive Fig. 2.** Tracing fluorescently labeled tCoa-NGR proteins *in vivo*. (A) Tumor-free mice injected with saline. Mice bearing PC3 prostate cancer xenografts injected with (B) FITC-labeled tCoa or (C) FITC-labeled tCoa-NGR (n=6).

**Supportive Fig. 3.** Therapeutic potential of tCoa-NGR fusion proteins *in vivo*. (A) Illustrative photos of mice bearing 4T1 solid tumors at the end of treatment (day 7) injected with tCoa-NGR (right) or tCoa (left). (B) Histological analysis of 4T1 and PC3 tumors in mice that were treated intravenously with saline, 10 µg tCoa or 10 µg tCoa-NGR (n=6). Arrows indicate thrombosed blood vessels. Magnification (×40)

**Supportive Fig. 4.** H&E and IHC analysis of PC3 tumor sections stained with CD13, Ki67, and CC3 in saline controls and groups treated with tCoa-NGR fusion proteins.
