# Supplementary figures and images for: NGR (Asn-Gly-Arg)-targeted delivery of coagulase to tumor vasculature arrests cancer cell growth
Source: Oncogene. 2018 Apr 17;37(29):3967–80. doi: 10.1038/s41388-018-0213-4 (PMC6053358; doi:10.1038/s41388-018-0213-4)

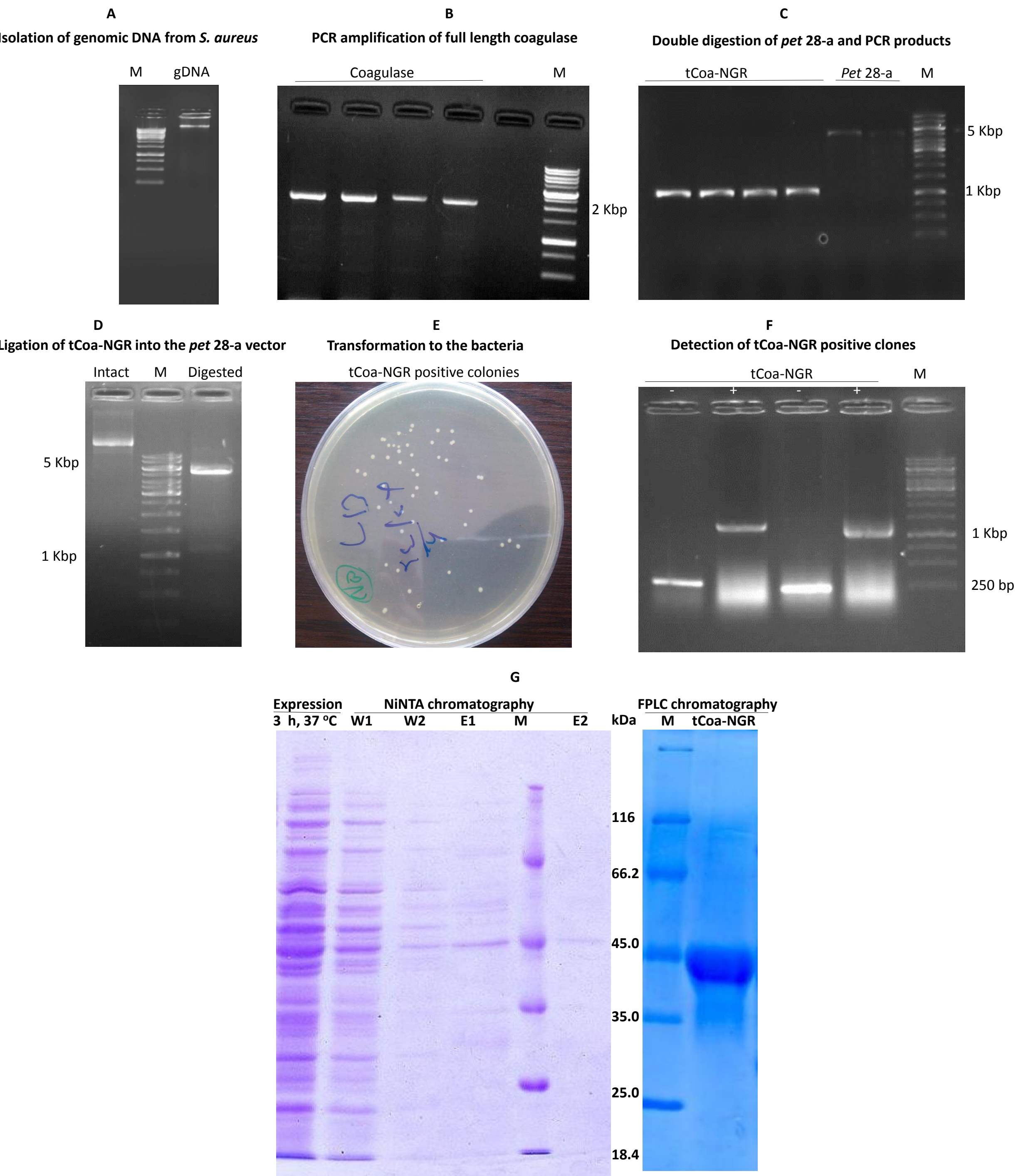

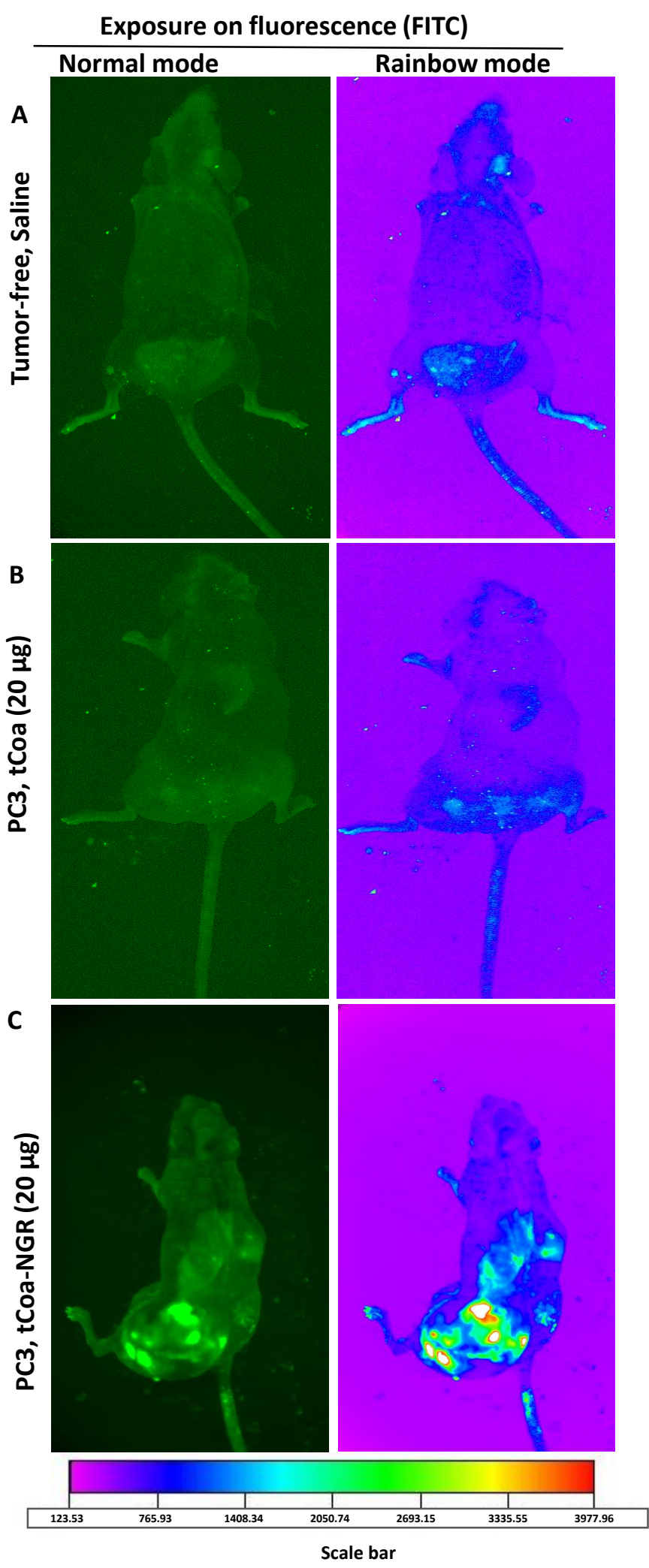

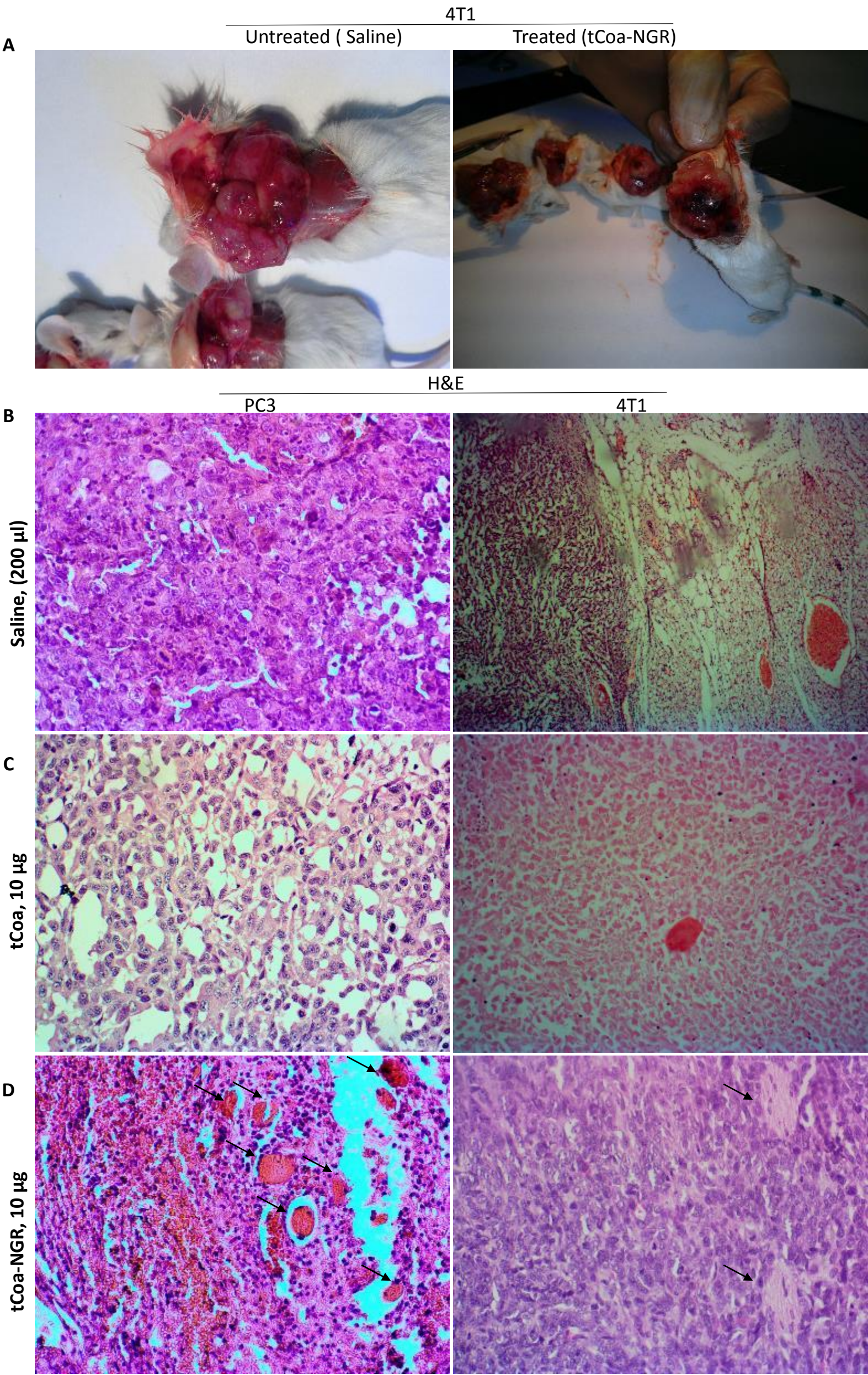

Untreated (Saline)

H&E

CC3

Ki67

CD13

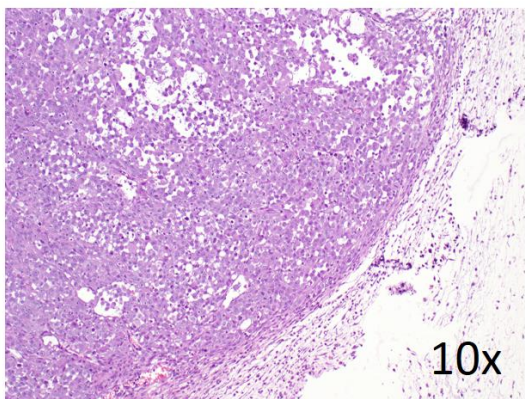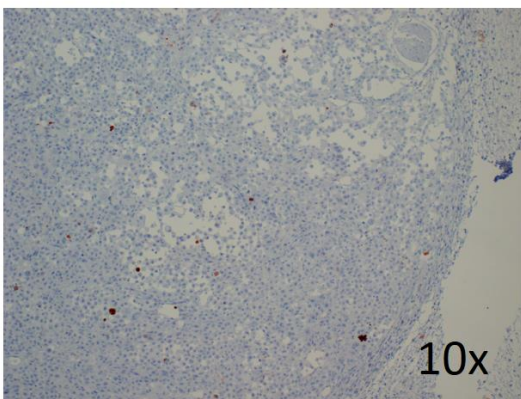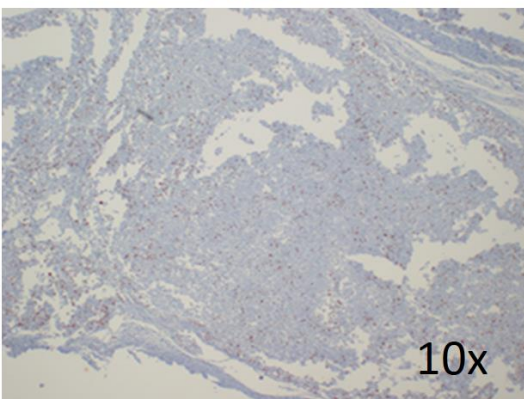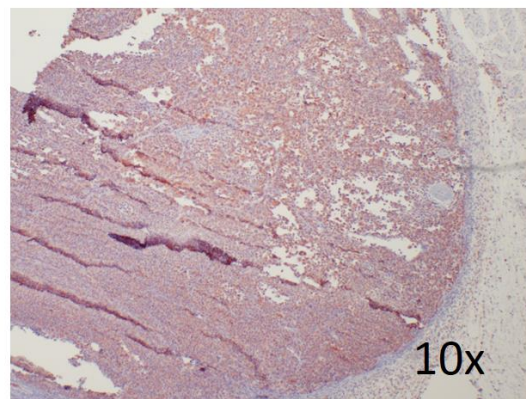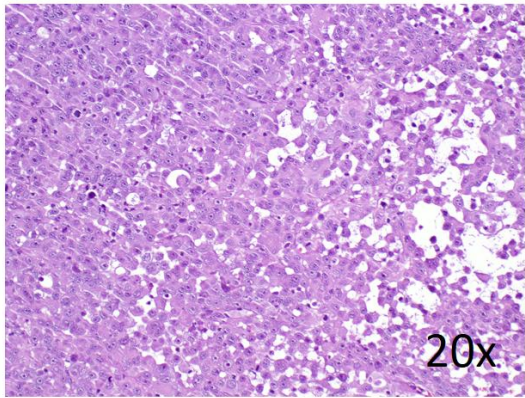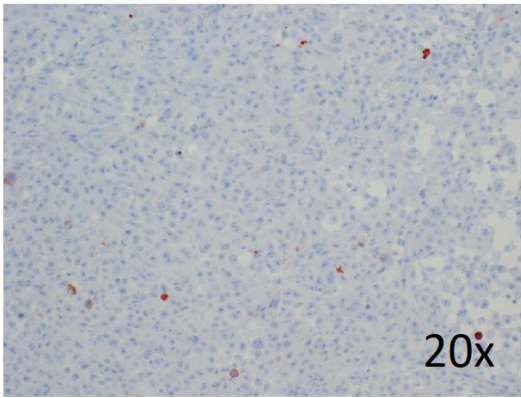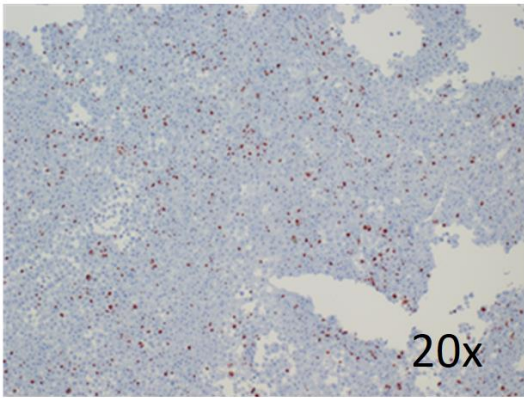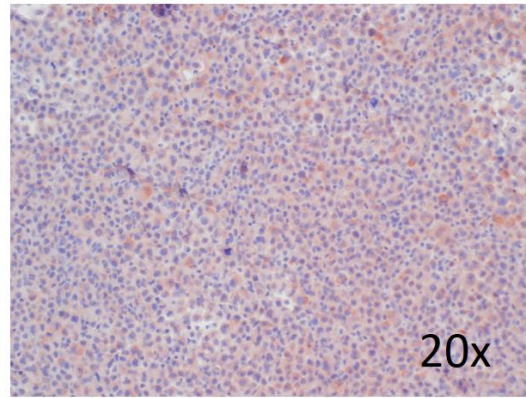

Treated (tCoa-NGR)

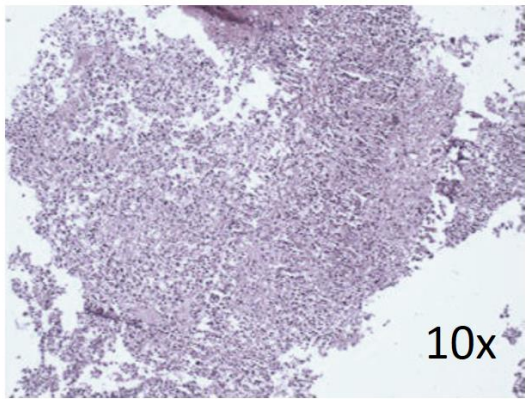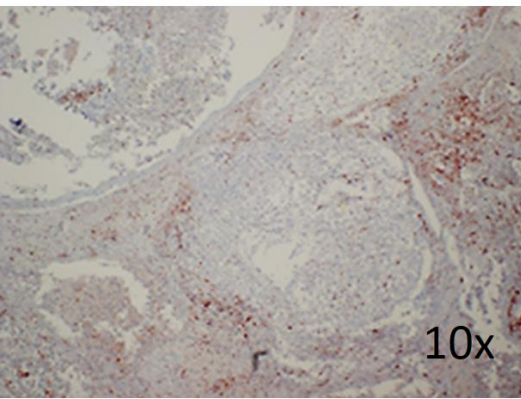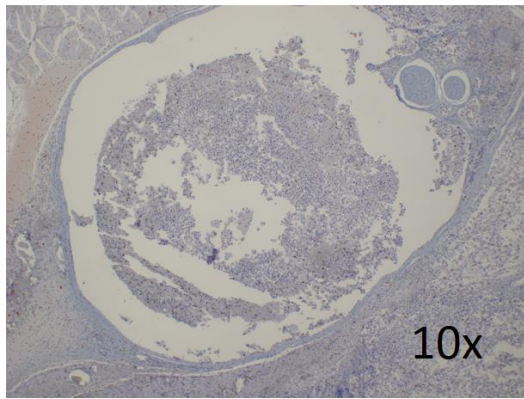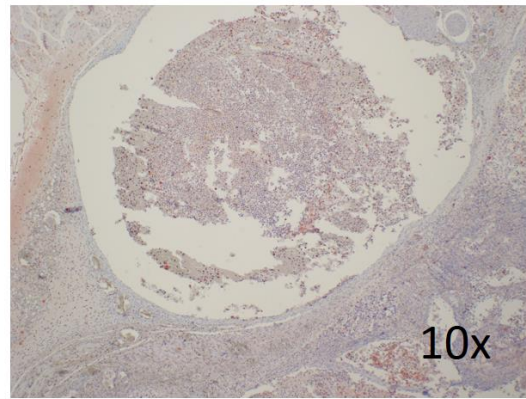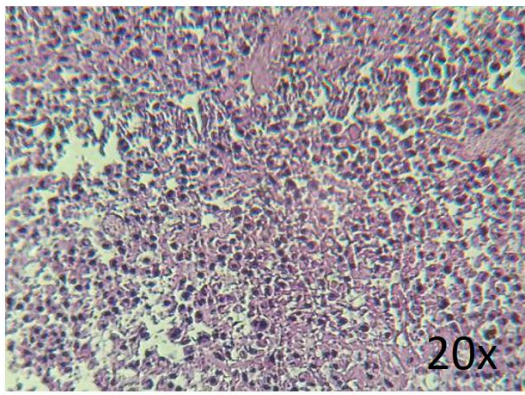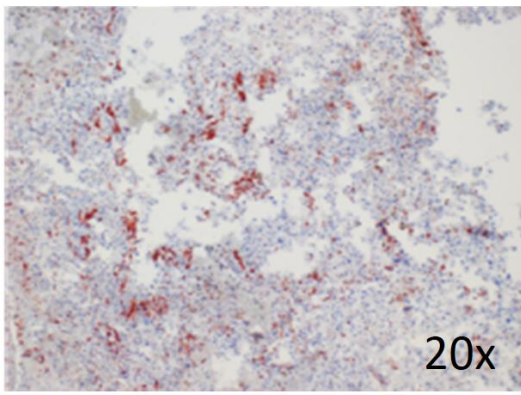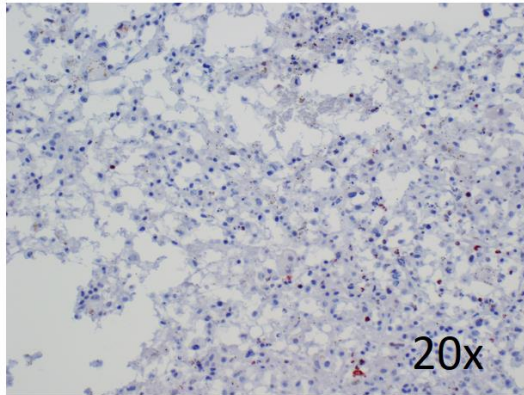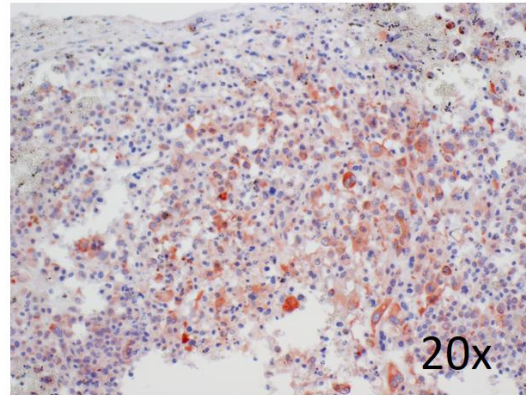

Supplement: Supplementary file 2 — Figs. S1–S4 [file 41388_2018_213_MOESM2_ESM.pdf]
